# Evolutionary characterization and transcript profiling of β-tubulin genes in flax (*Linum usitatissimum L*.) during plant development

**DOI:** 10.1186/s12870-017-1186-0

**Published:** 2017-12-08

**Authors:** Floriana Gavazzi, Gaia Pigna, Luca Braglia, Silvia Gianì, Diego Breviario, Laura Morello

**Affiliations:** Istituto Biologia e Biotecnologia Agraria, Consiglio Nazionale delle Ricerche, Via A. Corti, 12, Milan, 20133 Italy

**Keywords:** Flax, β-tubulin, Bast fibres, Expression analysis, Phylogenetic analysis, Gene family, Duplicated genes, Pollen tube

## Abstract

**Background:**

Microtubules, polymerized from alpha and beta-tubulin monomers, play a fundamental role in plant morphogenesis, determining the cell division plane, the direction of cell expansion and the deposition of cell wall material. During polarized pollen tube elongation, microtubules serve as tracks for vesicular transport and deposition of proteins/lipids at the tip membrane. Such functions are controlled by cortical microtubule arrays. Aim of this study was to first characterize the flax β-tubulin family by sequence and phylogenetic analysis and to investigate differential expression of β-tubulin genes possibly related to fibre elongation and to flower development.

**Results:**

We report the cloning and characterization of the complete flax β-tubulin gene family: exon-intron organization, duplicated gene comparison, phylogenetic analysis and expression pattern during stem and hypocotyl elongation and during flower development. Sequence analysis of the fourteen expressed β-tubulin genes revealed that the recent whole genome duplication of the flax genome was followed by massive retention of duplicated tubulin genes. Expression analysis showed that β-tubulin mRNA profiles gradually changed along with phloem fibre development in both the stem and hypocotyl. In flowers, changes in relative tubulin transcript levels took place at anthesis in anthers, but not in carpels.

**Conclusions:**

Phylogenetic analysis supports the origin of extant plant β-tubulin genes from four ancestral genes pre-dating angiosperm separation. Expression analysis suggests that particular tubulin subpopulations are more suitable to sustain different microtubule functions such as cell elongation, cell wall thickening or pollen tube growth. Tubulin genes possibly related to different microtubule functions were identified as candidate for more detailed studies.

**Electronic supplementary material:**

The online version of this article (10.1186/s12870-017-1186-0) contains supplementary material, which is available to authorized users.

## Background

Flax is an important crop, cultivated for both oil and fibre production. Bast fibres are phloem fibres characterized by an impressive cell elongation, up to several centimetres, and the deposition of gelatinous-type cell walls rich in cellulose, responsible for the particular tensile strength and flexibility of textile fibres. According to detailed studies performed by Gorshkova and co-workers [1, 2], different stem regions, corresponding to different fibre developmental stages, follow one another in fast growing flax plants. In the uppermost section, easily distinguished by smaller distance between leaves, most fibre cells elongate by coordinated growth, synchronously with the surrounding tissues and only the primary cell wall is present. Further downward, phloem fibres undergo intrusive growth by cell surface elongation. Finally, after elongation of bast fibres is completed, cell wall thickening takes place: the transition between cell elongation and cell wall thickening is marked by the snap point (SP), a region identified by the change in the mechanical properties of the stem, which becomes rigid and can’t anymore be easily broken by hand [2]. Downstream the SP, the galactan-enriched matrix (Gn-layer) of the secondary cell wall is gradually modified into a mature cellulosic gelatinous-layer (G-layer), showing the typical crystalline morphology of thickened cell wall, characterized by microfibril axial orientation, xylan absence, and a low content of lignin [3]. This change requires transcription of specific β-galactosidase genes (*LuBGAL1* and *LuBGAL2*) [4] and β-galactosidase activity, involved in remodelling of the cell wall matrix. Toward the base of the stem all phloem vascular bundles have completed cell wall thickening. Phloem fibre differentiation proceeds similarly during hypocotyl elongation, where the onset of secondary cell wall thickening follows cell elongation and is marked by an increase in β-galactosidase activity and up-regulation of corresponding genes along time [5]. For this reason, flax stems and hypocotyls have been used as a model to identify, through microarray studies, transcripts specifically expressed during phloem fibre differentiation [5, 6], and to study the expression of genes involved in cell wall development, such as glycosyl hydrolases (GH35) [7], cellulose synthase (CESA) [8, 9] and chitinase-like (CTL) genes [8].

Arrays of cortical microtubules (MT) control both the direction of cell elongation and cellulose deposition, fundamental processes related to fibre development. During bast fibre differentiation, as typical for diffusely elongating cells, MT orientation gradually changes from transversal, in the elongation stage, to longitudinal, re-orienting cellulose deposition at the onset of secondary cell wall thickening [1]. Cortical microtubules have a role in guiding CESA complexes from the trans-Golgi network to the plasma membrane and to drive microfibril orientation through the direct interaction with CESA complex [10]. Such interaction is mediated by cellulose-synthase interacting proteins (CSI) [11, 12]. It has also been reported that cellulose synthesis, defective in the *AtCesa2* mutant of Arabidopsis, can in turn affect microtubule reorientation [13]. MT also play a role in non-cellulosic polysaccharide assembly during cell wall biogenesis by recruiting vesicles carrying pectin and other cell wall matrix components to the cell cortex [14–17]. Finally, many signals triggering microtubule reorientation, such as auxin and light [18] were also known to differentially regulate transcription of α- and β-tubulin genes [19, 20]. This complex interplay between cell wall synthesis and cortical microtubule arrays might also involve specific tubulin isotypes. Tissue-specific expression of tubulin genes in plants has been widely reported [21] and up-regulation of particular tubulin isotypes has been related to specific cell functions, although direct evidence is limited. Overexpression of *EgTub1*has been reported to affect cellulose microfibril orientation in xylem fibres in *Eucalyptus* [22]. In *Populus*, genes encoding for xylem-abundant tubulins are also up-regulated in tension wood, which is highly active in cellulose deposition [23]. A subset of tubulin genes is preferentially expressed in elongating cotton fibres [24, 25]. One of these genes induces longitudinal growth when expressed in fission yeast [26]. Another plant structure characterized by polarized fast growth is the pollen tube. MTs play a role in secretion and endocytosis, acting as tracks for vesicular transport and deposition of proteins/lipids at the tip membrane [27]. Interestingly, overexpression of a *Picea wilsonii* pollen-specific alpha tubulin gene increases pollen tube growth rate in Arabidopsis [28]. Although such studies are not direct evidence for specific functions of particular tubulin isotypes, we can hypothesize that MT assembled from different tubulin pools, with slightly different aminoacid sequences, may show different kinetic or binding properties.

The peculiarity of flax fibres coordinated development, with a spatial separation of elongation and thickening along the stem axis and a temporal scansion during hypocotyls development, offers an opportunity to identify candidate tubulin genes involved in specific cell growth stages. In this manuscript, the large β-tubulin gene family of flax was phylogenetically characterized and the expression pattern of each member was investigated during flower development and along with stem and hypocotyl growth. Tubulin genes possibly related to cell elongation or cell wall thickening were identified as candidate for more detailed studies.

## Methods

### Plant growth conditions and tissue collection

Flax seeds (*Linum usitatissimum*, cultivar Valoal) were purchased from Semfor SrL. Plants were grown in pots with soil, in a growth chamber, under a 14 h light/10 h dark cycle, at 26 °C day/20 °C night, with 50% humidity. Stem tissues were collected during the fast growth stage, one month after sowing, from plants with similar height, 22 cm on average. The following sections were collected, after leaflet removal: apex (A) the uppermost 0,5 cm section; top section (TS) from 0,5 cm to 1,5 cm below the apex, above the snap point; medium section (MS) from 3,5 cm to 5,5 cm below the apex, encompassing the SP (located approximately at 4,5 cm from the apex), and basal section (BS), 2 cm long, about 2 cm upstream to the cotyledonary leaves. Male and female reproductive organs were collected from mature plants the day before anthesis (still unopened flowers with unelongated anther filaments) and early in the anthesis day (open flowers with elongated anther filaments and mature pollen). For hypocotyl collection, sterilized seeds were sown in magenta boxes with solid MS medium, kept in the dark for the first three days and then exposed to a 16 h light/8 h dark cycle, at 25 °C. Hypocotyls of similar length were sectioned at 3, 5, 7 and 12 days after sowing (DAS). For all the tissues, three independent samples of 100 mg each were immediately frozen in liquid nitrogen and stored at −80 °C.

### DNA extraction and capillary electrophoresis-TBP analysis (CE-TBP)

Genomic DNA was extracted from 100 mg of flax seedlings using the Qiacube® robotic workstation (Qiagen) with the DNeasy Plant Mini Kit (Qiagen). Extracted DNA was eluted in 150 μL Tris-EDTA buffer. DNA quality and quantity were assessed by NanoDrop-2000C spectrophotometer (Thermo Fisher Scientific). To generate the CE-TBP profiles, approximately 30 ng of genomic DNA was used as a template in TBP 1st and 2nd intron amplifications. The PCR reactions were performed in 30 μl according to [29]. Negative PCR controls (no template) were always included in each analysis. 4 μL of each PCR product were loaded on a 2% agarose gel to define the proper dilution rate for CE analysis. Capillary Electrophoresis was performed on the 3500 Genetic Analyser (Thermo Fisher Scientific) as described [30]. The data were collected using the Data Collection Software v. 2.0 (Thermo Fisher Scientific) and analysed by the GeneMapper Software v. 5.0 tool (Thermo Fisher Scientific).

### RACE amplification and cloning of the β-tubulin coding sequences

**Fig. 1:**
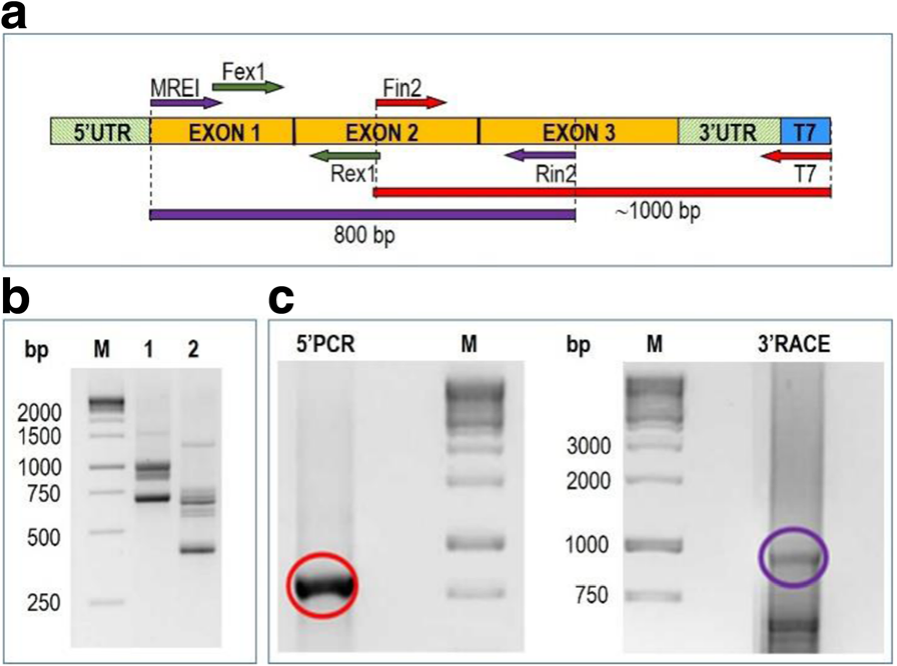
cDNA ends amplification of flax β-tubulin genes. (**a**) Cloning scheme with indication of primers and expected amplicons. (**b**) Test of the primer pair MREI/Rex1 on flax genomic DNA (lane 1) and Fex1/Rex1 control amplification (lane 2). (**c**) Agarose gel of 5′ and 3’ cDNA ends amplification

Five days-old frozen seedlings were ground in liquid nitrogen using a mortar and a pestle. Total RNA was extracted using the QIAcube (Qiagen) with the RNeasy Plant Mini Kit (Qiagen), according to the manufacturer’s protocol, starting from 100 mg of tissue and eluted in 50 μl of nuclease-free water. To verify RNA integrity, 2 μL of each sample were loaded on 1% (*w*/*v*) agarose gel and stained with ethidium bromide. RNA concentration and purity were assessed spectrophotometrically. RNA samples were treated with an RNase-free-DNase I (Thermo Fisher Scientific) at 37 °C for 30 min and stored at −80 °C until use. Single strand cDNA was synthesized starting from 1 μg of total RNA, using the T7-tagged primer T7(dT)25 and 1 μL (200 U) Superscript II (Invitrogen) in the presence of RNase OUT (Thermo Fisher Scientific). After RNA digestion with RNase H, 5′ amplification and 3’ RACE were set up in parallel, by targeting respectively the cDNA 3’end with Fin2/T7 and the 5’end using MREI/Rin2 (Additional file 1), as depicted in Fig. 1a. PCR was performed using 1 μL cDNA, with 0,6 U Hot Start Taq Polymerase Advance I (Clontech), in 20 μL volume, with the following thermal profile: 94 °C, 3 min; 14 cycles of 94 °C 30 s, 56 °C 45 s, 72 °C 2 min; followed by 20 cycles in which the annealing temperature was decreased by 0.5 °C at each cycle and 10 min at 72 °C for final extension. The resulting amplicons were cut from gels, eluted using the NucleoSpin Extract II Kit (Macherey-Nagel) and cloned into the pCR4-TOPO TA cloning vector (Invitrogen). Positive clones were sequenced on both strands and sequences from both 5′ and 3’ RACE were assembled using Contig Express, the assembly tool of the NTI Vector Suite (Invitrogen). Assemblies were manually cured to resolve ambiguities and 14 contigs were generated. To confirm contig sequences, full-length cDNA clones were obtained by amplification with the degenerate MREI and Reverse Specific Primers (RSP, Additional file 1) designed on the most 3’terminal region of each contig. Cloning and sequencing gave the correct, final cDNA sequences, starting from the beginning of the ORFs.

### Gene expression by qRT-PCR

Gene expression was studied in stem, hypocotyl and flower samples collected as described in the previous section. Total RNA was extracted from frozen samples as described for RACE. cDNA was obtained using the Revert Aid H Minus First Strand cDNA Synthesis Kit (Thermo Fisher Scientific), according to the manufacturer’s protocol, starting from 1 microgram of total RNA. Samples were finally treated with RNase H (Thermo Fisher Scientific) 0,1 U/ μL, at 37 °C for 20 min and were stored at −20 °C.

Isotype-specific primer pairs, suitable for qRT-PCR, were manually designed in the 3’UTR regions and evaluated with the help of the Primer Express software (Invitrogen). Primer specificity was evaluated by BLASTn search in the flax WGS database and confirmed in real-time PCR by re-association kinetics. *LuBGAL1* (GenBank: HQ902252.1) primers were from [4]. Specific primers for reference genes *Elongation Factor 1-α* (*EF1α*) and *Glyceraldehyde-phosphate dehydrogenase* (*GAPDH*) were from [31]. All qPCR primer sequences are listed in Additional file 1.

A five-point standard curve of a 4-fold dilution series (1:4 to 1:1024) of a cDNA mix from various tissues, was used to calculate the PCR efficiency of each primer pair and the optimal cDNA dilution for quantitative analysis (Ct comprised between 20 and 28). For the expression analysis, 1,5 μL of a 1:16 dilution of each cDNA sample was used. qRT-PCR reactions were performed in 96 well plates, in a CFX Connect Real Time System (BioRad), in a final volume of 20 μl containing 1,5 μL of cDNA, 0,4 μM of each primer and 10 μL of 2× Sso Advanced TM Universal SYBR® Green Supermix (BioRad), under the following conditions: 95 °C for 3 min, 40 cycles of 15 s at 95 °C and 60 s at 62 °C. A 65–95 °C melting curve was performed to confirm the specificity of the products.

Each experiment was repeated twice on two biological replicates (RNA extraction and cDNA synthesis), with three technical repetitions each. Data analysis was performed using the BioRad CFX Manager Software. Threshold was manually set at the same level to compare results from different plates. Correction for specific amplification efficiency and inter-run variation were applied in Gene Studies. Ct values were normalized using the geometrical mean of *EF1α* and *GAPDH* as reference genes. Stability acceptance values (CV < 0.5; M < 1) for both reference genes were respected [32]. The ΔΔCt method with Pfaffl correction for amplification efficiency [33, 34] was used to compare transcript fold increase, relative to zero (mean expression of each target) or to a control sample. Heat maps were generated using the same BioRad CFX Manager Software. Each data point represents the expression level of one target sequence in one sample, relative to the average of the reference genes. Data points are scaled by target and clustered by the degree of similarity of expression.

### Sequence analysis

Cloned cDNA sequences were assembled and aligned using the Contig Express and Align X tools of the NTI Vector v.11 suite and manually refined. Phylogenetic analysis was performed using MEGA5 [35]. Deduced amino acid sequences were aligned by MUSCLE with default settings. The phylogenetic analysis was performed choosing the “best substitution model” tool provided by the software. The chosen model (Jones Taylors-Thornton, JTT by Gamma distribution rate with Invariant sites, G + I) was applied to infer a phylogenetic tree by the Maximum Likelihood (ML) estimation method. The initial tree was inferred by Neighbour Joining analysis, following the Nearest-Neighbour-Interchange heuristic method. The statistical confidence of a particular group of accessions in the tree was evaluated by bootstrap test with 1000 replicates. The tree was rooted using *Chlamydomonas reinhardtii* β-tubulin as the outgroup. Accession numbers of all aminoacid sequences are provided in Additional file 2. Schematic drawing of the exon-intron structure of tubulin genes was obtained by alignment of the cDNA and genomic sequences in the Gene Structure Display Server website tool, v 2.0 (GSDS, http://gsds.cbi.pku.edu.cn, accessed 20 October 2016).

## Results

### PCR amplification and cloning of the β-tubulin genes

To isolate simultaneously the coding region of all members of the β-tubulin gene family, we designed an amplification strategy based on degenerated primers effective on all plant β-tubulin genes. This is made possible because of the strong evolutionary conservation of tubulin coding sequences, due to the extraordinary structural constraints imposed to tubulin by its fundamental functions. Short nucleotide stretches, showing 70–80% overall identity, are useful to design slightly degenerated universal primers. Internal primer pairs Fex1-Rex1 and Fin2-Rin2, annealing to the ORF at the first and second intron borders respectively, have been abundantly tested because they are at the base of the genomic profiling method called tubulin-based polymorphism (TBP), exploiting on the different length of β-tubulin introns [29, 36]. A further, degenerated forward primer, named MREI, was designed at the very 5′ end of the β-tubulin open reading frame, where the first six aminoacid positions (M-R-E-I-L-H) are extremely conserved among all plant β-tubulins. RNA extracted from 5 days old seedlings was used as a template for cDNA synthesis with a T7-tagged primer. 5′ and 3’ cDNA regions were independently amplified with MREI/Rin2 and Fin2/T7 primer combination to generate two overlapping cDNA portions (Fig. 1a).

The degenerated MREI primer was first tested on flax genomic DNA. In combination with the reverse primer Rex1, it revealed a band pattern shifted in size by 300 bp with respect to a control amplification done with the forward primer Fex1, commonly used in the CE-TBP profiling protocol (Fig. 1b). As predicted, cDNA amplification of the 5’terminus of the ORF, spanning from the start codon to the middle of the third exon, originated a unique sharp band on agarose gel, since all plant β-tubulin cDNA have the same length in this region (Fig. 1c). Conversely, 3’RACE produced a smeared band due to the heterogeneous length of the third exon and the 3’UTR of β-tubulin genes, together with some shorter, non-specific amplicon (Fig. 1c). Amplified bands were eluted from gel and cloned into *E.coli* for sequencing. After nucleotide sequence assembly, tentative contigs were obtained for 14 different β-tubulin cDNA sequences, spanning from the beginning of the coding sequence to the poly-A tail. Contig accuracy was confirmed by cloning and sequencing of fourteen different cDNA fragments amplified with the MREI forward primer and Reverse Specific Primers (RSP), designed at the most downstream position of each 3’UTR (Additional file 1). cDNA sequences ranged in length from 1463 to 1665 nucleotides.

### The flax β-tubulin gene family

Alignment of the cDNA sequences revealed that the 14 β-tubulin genes were highly similar two by two (three in one case), generating pairs of identical or nearly identical proteins, plus one singleton. Because of this feature, we assigned the same number, followed by letters a, b or c, to the duplicated genes. Hereafter the name of the gene followed by the number will refer to both duplicated genes. Such duplicated genes are the likely result of a whole-genome duplication occurred in flax 5–9 Ma ago (MYA), before the speciation event leading to *Linum bienne*, the wild ancestor of *Linum usitatissimum* [37]. We will therefore refer to these duplicate tubulin genes as ohnologs, according to Wolfe’s suggestion for autopolyploid paralogs [38]. Labels assigned to the 14 cDNA β-tubulin sequences, accession numbers and other sequence information, are listed in Table 1 (columns 1 to 9), together with the accession numbers of the putative genomic loci (column 10) and the corresponding cDNA as annotated in the Phytozome database (column 11). Genomic sequences were retrieved from GenBank (https://blast.ncbi.nlm.nih.gov, accessed 12 September 2016), through a BLAST search against the first assembly of the flax Whole-Genome Shotgun Sequencing (release 1.0, [37]), using as query the more variable regions, close to the 3′-end of each of our 14 transcripts. Individual alignments between our cDNA sequences and the corresponding best hits revealed some mistake in the genome assembly. In fact, genomic sequences most similar to our *LusTub2a*, *7b* and *6a* cDNA were actually hybrid sequences likely derived from misassembly of the highly similar sequences of the two ohnologs. The genomic counterpart of our *LusTub7b* contained a 22 bp insert causing an improbable reading frameshift that is not present in our cDNA clones. Moreover, *LusTub7c* aligned only with a short, partial, tubulin-coding sequence at the end of one scaffold. However, ESTs sequences bearing a 3’terminus perfectly matching *LusTub7c* were retrieved from GenBank, confirming the existence of three similar but different *LusTub7* loci in the flax genome. As a consequence of the missing sequence, only 13 putative transcripts, annotated as coding for β-tubulin-like chains, were found in Phytozome, Version 11 (https://phytozome.net, accessed 13 September 2016): the partial sequence corresponding to the *LusTub7c* cDNA was not present.

**Table 1:** List of *Lusb-tub* cDNA and corresponding genomic sequences

			Sequence length (pb)							
Gene name	cDNA (GenBank ID)	Class	ORF length (excluding stop)	Intron 1	TBP amplicon 1	Intron 2	TBP amplicon 2	Protein length (AA)	Putative genomic locus (GenBank ID)	Putative transcript (Phytozome ID)
*LusTub1a*	KM196480	II	1347	428	733	508	740	449	AFSQ01023152	Lus10027476
*LusTub1b*	KM196481	II	1347	399	704	530	762	449	AFSQ01018519	Lus10039231
*LusTub2a*	KM196482	I-like	1347	109	414	188	420	449	AFSQ01019209^a^	Lus10035497
*LusTub2b*	KM196483	I-like	1347	104	409	188	420	449	AFSQ01017984	Lus10002000
*LusTub3a*	KM196484	III	1335	983	1288	104	336	445	AFSQ01016600	Lus10021094
*LusTub3b*	KM196485	III	1335	959	1264	103	335	445	AFSQ01010333	Lus10017217
*LusTub4a*	KM196486	IV	1326	272	577	740	972	442	AFSQ01008040	Lus10023348
*LusTub4b*	KM196487	IV	1326	299	604	506	738	442	AFSQ01002817	Lus10038458
*LusTub5*	KM196488	I-like	1345	103	408	727	959	448	AFSQ01013709	Lus10008528
*LusTub6a*	KM196489	I	1338	353	658	93	325	446	AFSQ01000745^a^	Lus10036069
*LusTub6b*	KM196490	I	1344	359	664	85	317	448	AFSQ01016220	Lus10026813
*LusTub7a*	KM196491	II	1350	96	401	99	331	450	AFSQ01023518	Lus10040712
*LusTub7b*	KM196492	II	1350	101	406	98	330	450	AFSQ01004919^a^	Lus10016448
*LusTub7c*	KM196493	II	1350	?	?	?	?	450	AFSQ01023517^b^	not found

^a^hybrid seq

^b^partial seq

**Fig. 2:**
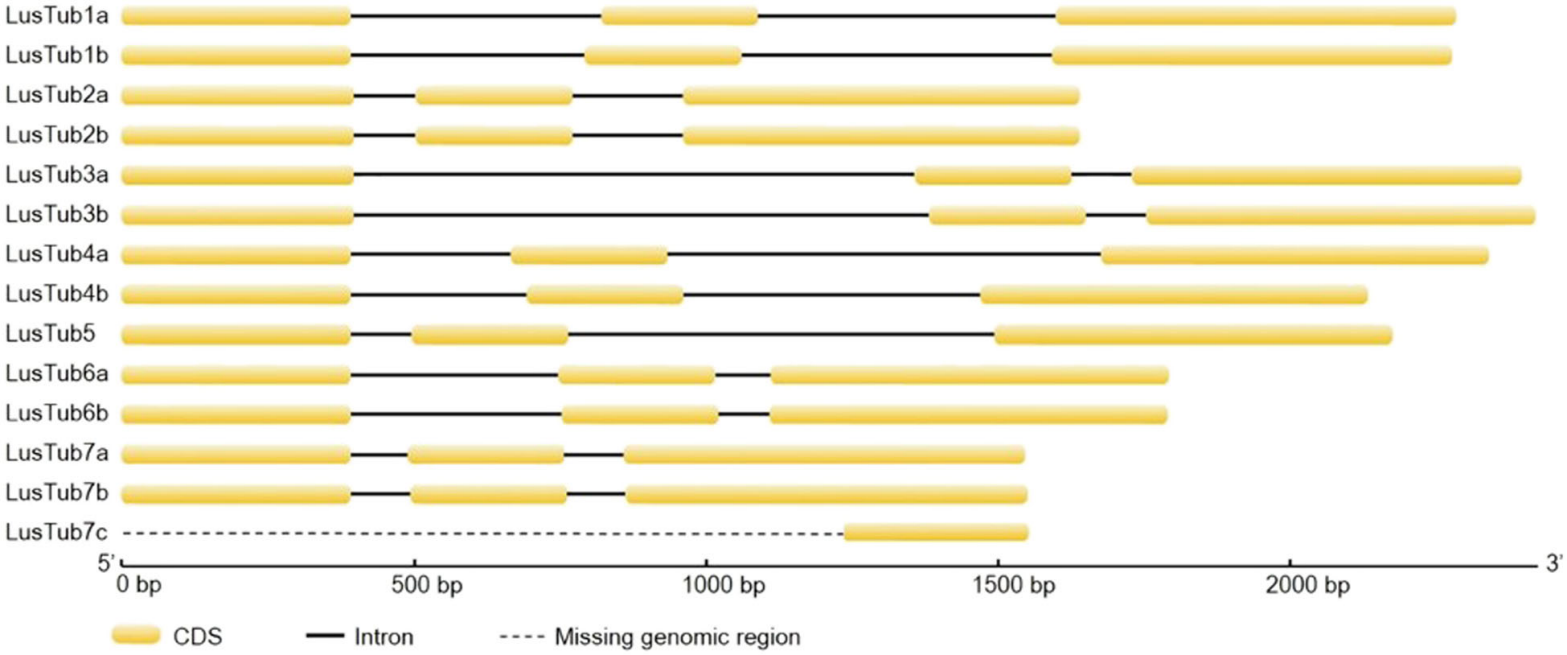
Exon/intron structure of flax β-tubulin genes. cDNA sequences were aligned to their putative genomic counterparts, listed in Table 1. Only a short sequence corresponding to *LusTub7c* was found in the NCBI database (dotted line indicates the missing part)

All described β-tubulin genes of land plant possess the same conserved structures with three exons and two introns at highly conserved positions, with rare exceptions represented by the loss of the second intron as in *Zea maysTUB1* and *Oryza sativaTUB2* [39]. Alignment of flax cDNA with genomic β-tubulin sequences confirmed this exon/intron organization, with very little variability in intron lengths among ohnologs (Fig. 2, Table 1). The only noticeable exception was that of *LusTub4a*, whose second intron was clearly longer than that of its *LusTub4b* counterpart (740 vs 506 nucleotides).

### Accordance of the putative β-tubulin gene family with the TBP profile

**Fig. 3:**
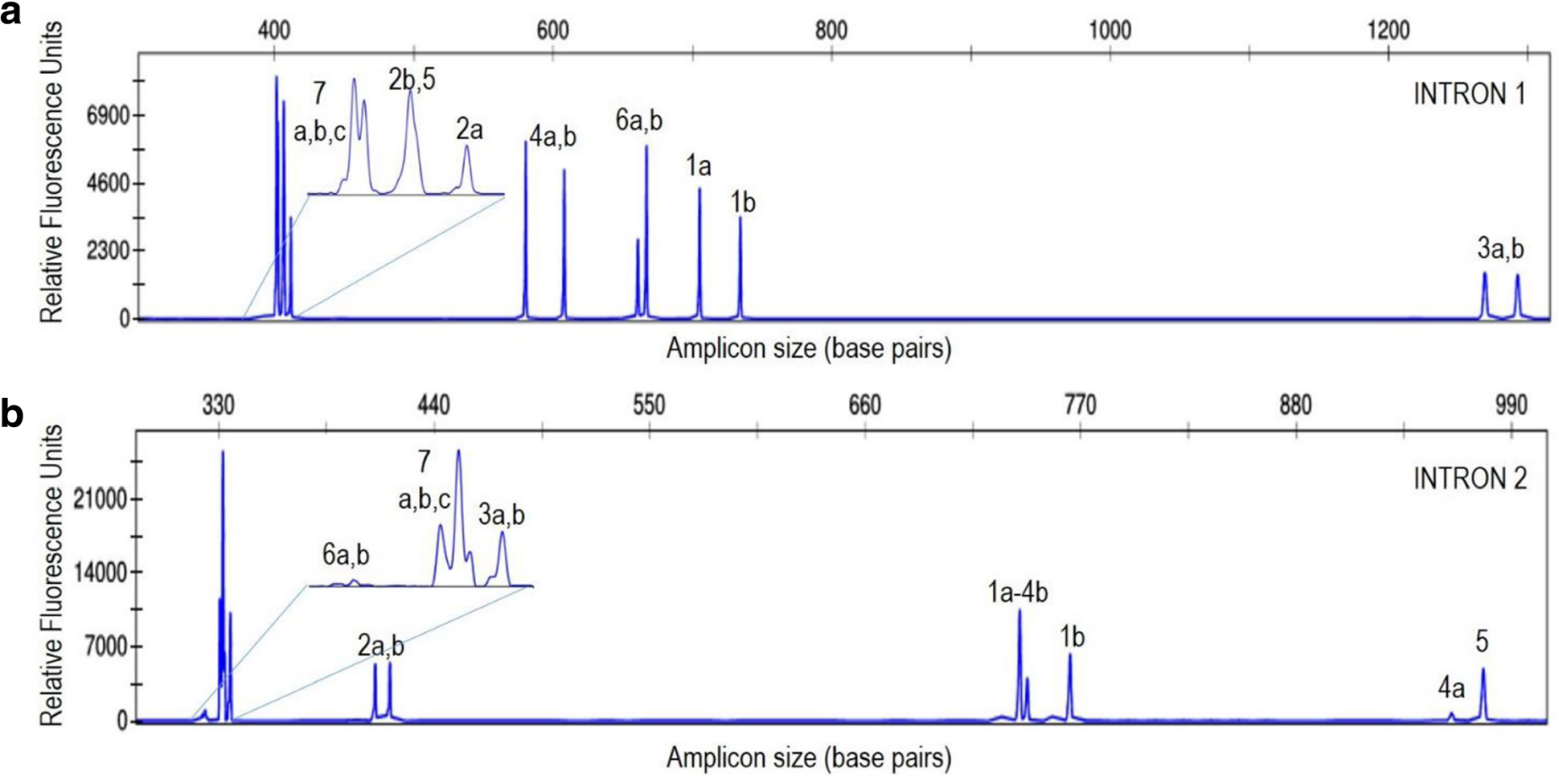
CE-TBP profiling of flax β-tubulin introns. (**a**) first intron. (**b**) Second intron. Insets: magnification of the short size amplicon peaks

To confirm that the 14 transcripts represented the complete set of the β-tubulin genes, we analyzed the flax genomic DNA with the CE-TBP method [40], capable of producing a reliable and an effective profiling of the β-tubulin gene family through Exon-primed Intron crossing. The TBP protocol was originally set up in our laboratory as a useful marker for the fast genotyping of plants at both species and variety level. In fact, The TBP-PCR multiplex amplification triggered by the Fex1-Rex1 and Fin2-Rin2 primer combinations, generates two sets of amplicons producing specific profiles once separated capillary electrophoresis. Each profile is representative of the number of paralogous genes, variable from 7 to more than 20 across different plant species, and the length of the first or the second intron of β-tubulin genes. TBP profiling thus provides a fast and convenient way to evaluate the numeric consistency of the gene family [30]. Profiles of both introns in the flax genome were perfectly consistent with the expected number and size as retrievable from the genomic sequences (Fig. 3, Table 1). This seem to exclude the existence of other undetected β-tubulin genes or pseudogenes in the flax genome.

### Phylogenetic analysis

**Fig. 4:**
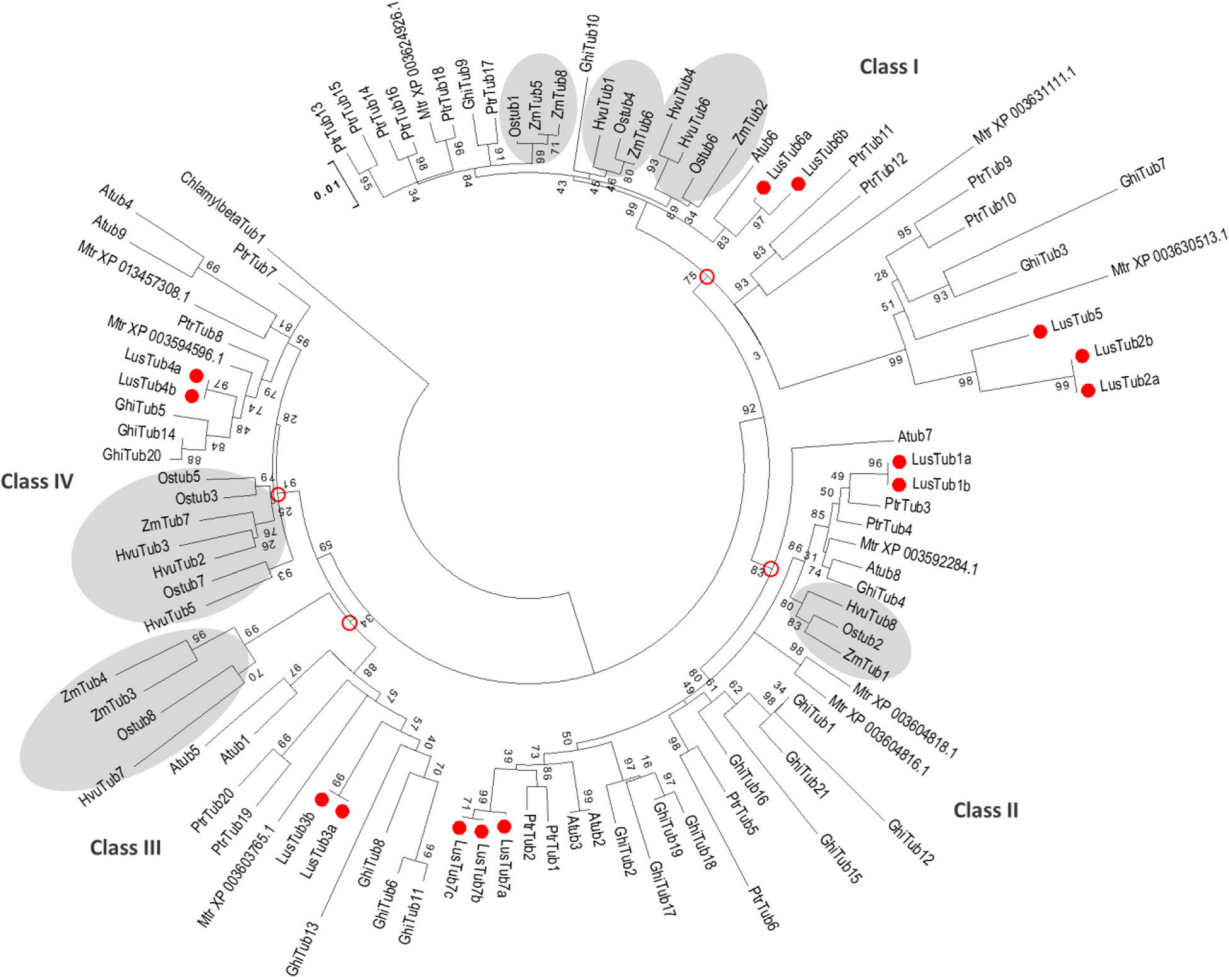
Similarity tree of plant β-tubulins. Maximum likelihood analysis was performed on the complete β-tubulin sets of flax, aspen, cotton, Arabidopsis, *Medicago*, barley, rice and maize. Accession numbers are listed in Additional file 2. The β-tubulin of the green alga *C. reinhardtii* was used as an outgroup. Close circles indicate flax tubulins. Open circles indicate the nodes of the four tubulin classes. Grey background indicates monocot tubulin clades

**Fig. 5:**
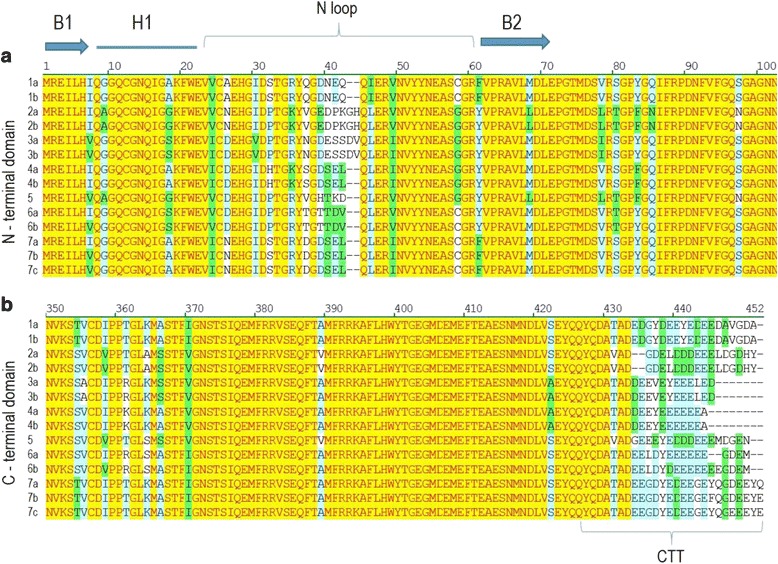
N-terminal and C-terminal sequence alignment of flax β-tubulins. Aminoacid sequences were aligned with Clustal W. Background color code: Yellow: identical; green: block of similar; light blue: conserved; white: non-similar. B: beta-sheet; H: alpha helix. (**a**) Amino-terminal region, from residues 1 to 103. (**b**) Carboxy-terminal region, from residue 350 to 452. CTT: C-terminal tail

When aligned to all the 74 β-tubulins from 7 other angiosperms (3 monocots and 4 dicots), listed in Additional file 2, the 14 flax deduced aminoacid sequences clustered within four monophyletic clades in the maximum likelihood phylogenetic tree of Fig. 4. Five members belong to class I (LusTub 2a, 2b, 5, 6a and 6b), five members to class II (LusTub 1a, 1b, 7a, 7b, 7c), two members to class III (LusTub 3a and 3b) and two members to class IV (LusTub 4a and 4b). The clade including LusTub 2a, 2b and 5, is characterized by greater branch length, reflecting a higher number of aminoacid substitutions occurring in these sequences with respect to the core Class I group. This clade, merely including few dicot representatives, was considered as a fifth class, referred to as Class I-like [23], Class V [21] or Class 2 [41], in previously published similarity trees. Our phylogenetic reconstruction based on the ML method, using a large dataset (88 samples) and complete sampling within each species, strongly supports the existence of just four classes (nodes are indicated by red circles in Fig. 4) and favours the positioning of former Class I-like/ClassV/Class 2, as a diverging subclass within Class I. An even smaller and divergent cluster, just including two aspen and one *Medicago* β-tubulins in our tree, also forms a sister clade within class I. These two small subclasses include at present only dicot members, possibly related to exclusive features of this lineage. Monocot tubulins are present in each of the four classes throughout the tree, although forming separate clusters (grey background in the tree): this observation suggests that all the extant β-tubulin genes of flowering plants are the descendants of four ancestral genes, predating the separation of monocots and dicots from a common ancestor. Although sequence features allow a clear distinction of the different plant β-tubulin classes (i.e, most Class III members show additional AA in the N loop), no functional difference have been demonstrated for any class or isotype yet [39]. Due to the conservative nucleotide substitutions between ohnolog pairs, the 14 β-tubulin genes produced only 10 different proteins. Protein length spanned from 442 to 450 aminoacids, with an overall sequence identity of 85.8%. Most difference was concentrated in the C-terminal tail (CTT), spanning from AA 434–450 (Fig. 5). Outside this domain, variable positions were only 45 out of 433, most of which clustered at the N-terminal domain, in particular within the H1-S2 loop, also called the N-loop, between AA 33–44, and few others in a downstream region between AA 354–365 (Fig. 5). At position 40 in the N-loop, most plant β-tubulins, as well as all vertebrate β-tubulins, lack two AA residues if compared to α-tubulins. Exception is provided by LusTub3a and 3b in flax, both bearing the two missing aminoacids, and by most members of the same class III in other plant species (i.e. AtTUB1, and TUB5, GhTUB6, 8, 11 and 13), some of which bear even one or two additional residues [25, 39]. An interesting finding in flax is the unique occurrence of two additional AA in the N-loop of tubulin LusTub2a and 2b, both belonging to Class I. The role of such AA variability at the N-loop in plant β-tubulins has been very poorly investigated and its contribution to peculiar properties of the heterodimer might go underestimated. In fact, according to the tubulin dimer structure model [42], the N-loop interacts with the M loops of laterally adjacent subunits. Recent homology modelling studies, based on the 1JFF for β-tubulin of *Bos taurus*, showed that additional AA in this region of β-tubulin contribute a higher stability to microtubules [43]. Flax β-tubulins from ohnologous genes diverged slightly, with aminoacid substitutions ranging from zero (LusTub1, 2, 3 and 4) to 4–5 (LusTub 6 and 7), mostly concentrated in the CTT.

### Expression profiles along fibre differentiation

β-tubulin gene expression was studied by RT-qPCR in stem sections at increasing distance from the apex and in hypocotyls at different days after sowing, taken as model systems for the progression of fibre differentiation. Stems of 30 days old plants (fast growth stage) were deprived of leaves and sectioned in order to distinguish three different phases of phloem fibre growth, according to what reported in published studies [2, 6]. 1) The top section (TS), easily distinguished by short internodes, characterized by fast elongating phloem cells with primary cell wall and transversally oriented cortical MT; 2) the medium section (MS), around the SP, characterized by the gradual cessation of fibre cell elongation and the onset of secondary cell wall thickening, with a prevalence of helical arrays of cortical MT, due to their gradual reorientation; 3) the basal, or bottom section (BS), close to the base of the stem, containing fully differentiated fibres, with thickened, gelatinous type cell walls and longitudinally oriented MT. The apical section (A), which includes the apical meristem with actively dividing and early differentiating cells, was analysed as a control. In elongating hypocotyls, cell division has almost ceased during the pre-germinative stage and fibre differentiation proceeds similarly to the stem, with cell elongation preceding in time secondary cell wall deposition and thickening [44]. Hypocotyls were collected at four different stages, DAS 3, 5, 7 and 12, toward the end of elongation. This study provided an overview on the modulation of β-tubulin gene expression during plant growth, useful to identify some interesting candidate for further investigations on fibre differentiation.

Due to the very high degree of sequence similarity between ohnologs, gene-specific primers were designed in the 3’untranslated regions of each tubulin cDNA (Additional file 3). Expression of the *β-Galactosidase 1* gene (*LuBGal1*), known to be up-regulated during cell wall galactan formation in fibre cells [4, 6, 45], was used as a marker for the fibre developmental stage in both the stem and hypocotyl.

**Fig. 6:**
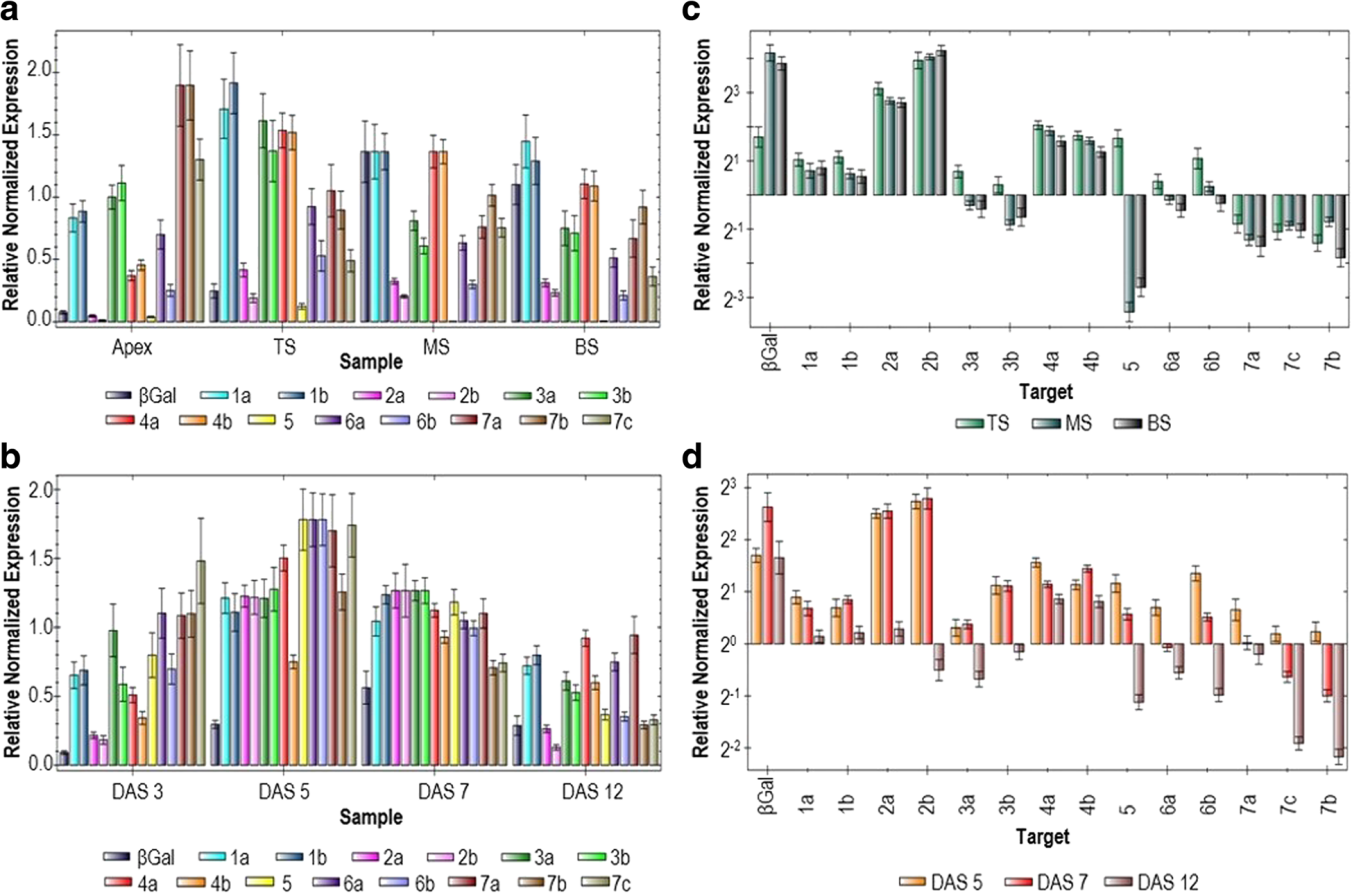
Relative differential expression of flax β-tubulin genes in stem sections and hypocotyls. Relative transcript levels of the 14 flax β-tubulin genes, normalized to the geometric mean of two reference genes, as evaluated by real-time qPCR in stem sections (**a** and **c**) and hypocotyls (**b** and **d**). Expression levels, grouped by sample and relative to the avarage expression of each target, are shown in panels **a** and **b**. In panels **c** and **d**, transcript amounts are grouped by sample and normalized to a control sample: the apex in panel **c** and the DAS 3 sample in panel **d**. A Log2 scaling was used for the y axis

Normalized gene expression was plotted by sample type for the stem (Fig. 6a) and hypocotyl (Fig. 6b), showing that all β-tubulin genes were transcribed in both organs, although at different levels, during fibre differentiation. In the stem, the transcript profile of the apex, with *LusTub7* predominantly expressed and *LusTub2* and *5* transcripts barely detectable, greatly differed from that of TS, where fast fibre cell elongation was accompanied by a generalized increase in β-tubulin transcript levels, with *LusTub1*, *3* and *4* becoming the prevalent transcripts, and by the down-regulation of the *LusTub7* group. The transition from fibre elongation to cell wall thickening, clearly marked by the increase in the *LuBGal1* transcript from the top to the medium section, was mainly characterized by the down-regulation of *LusTub3* genes and the complete disappearance of the *LusTub5* transcript. This variation was concomitant with MT re-orientation from transversal to longitudinal, characterizing the arrest of cell elongation [1]. No relevant variation of the expression pattern was observed in the next section, BS, characterized by complete cell wall thickening in fibres. In the hypocotyl, the transition from cell elongation to cell wall thickening in fibres, marked by *LuBGal1* up-regulation, could be mapped at the DAS7 stage in our system, while major variation in tubulin gene expression was observed earlier, between DAS3 and DAS5, during the cell elongation stage, when all β-tubulin genes were up-regulated, although at different extent. DAS5 and DAS7 did not show relevant modification of the relative transcript ratio, while a more varied pattern was observed at DAS12, whose profile was similar to that of stem MS and BS. The main difference between the two plant organs was represented by *LusTub5* transcript, barely detectable in the stem, but highly expressed in the hypocotyl.

Comparison of the same data, reported as fold changes of each target gene with respect to the apex in the stem and to DAS3 in the hypocotyl, highlighted similarity in the modulation of the β-tubulin gene expression pattern, despite different absolute levels (Fig. 6c and d). Genes that were more stably up-regulated during fibre growth in the stem, *LusTub1*, *2* and *4*, were similarly up-regulated in the hypocotyl. *LusTub5*, *3* and *6*, transiently up-regulated during the elongation stage, were progressively down-regulated as cell wall thickening progressed. *LusTub7* transcript level decreased in both organs with respect to the apex or the 3-DAS stage, in parallel with fibre elongation and differentiation. This observation is in agreement with a strictly controlled expression of β-tubulin genes during stem and hypocotyls elongation, possibly related to fibre differentiation.

### β-tubulin gene expression in reproductive organs

**Fig. 7:**
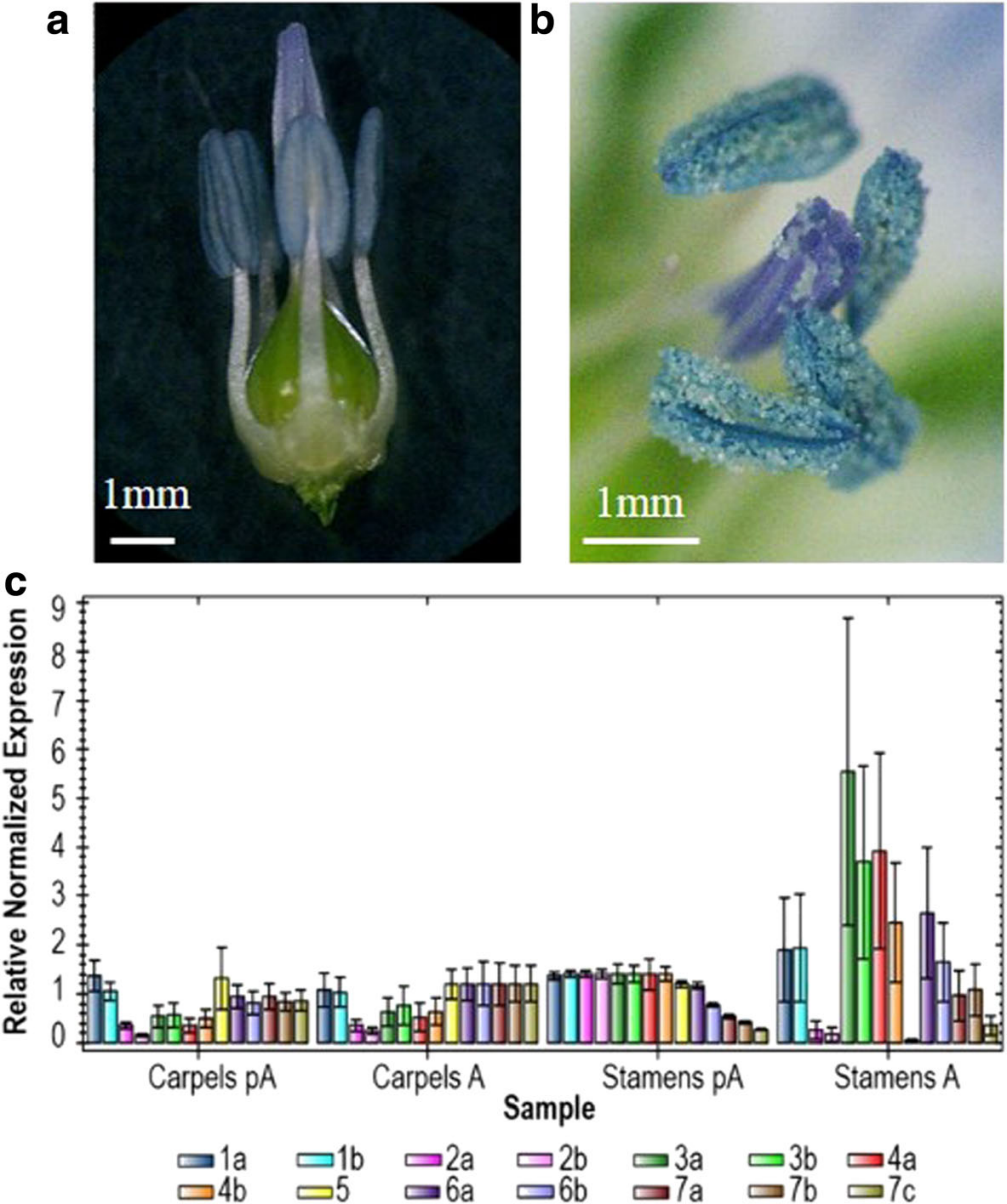
Relative differential expression of flax β-tubulin genes in reproductive organs. Flax reproductive organs were collected the day before anthesis (**a**) and the day of flowering (**b**) and observed under a stereomicroscope. Scale bar: 1 mm. **c**, relative transcript level of the 14 flax β-tubulin genes in stamens and carpels collected the day before anthesis (pA) and the day of anthesis (**a**) were measured by RT-qPCR and normalized to the the geometric mean of two reference genes

Plant reproductive organs often show a prevalent expression of specific tubulin isotypes. In particular, mature pollen is characterized by the preferential if not exclusive expression of specific alpha and beta isotypes in both maize and Arabidopsis [46–49] and this might relate to pollen tube growth. In order to identify interesting candidates for further investigation, we analysed β-tubulin gene expression in male and female organs of the flower either the day before anthesis, corresponding to stage 11 of flax flower ontogenesis as defined by [50] (Fig. 7a), or the day of anthesis, when flowers open and the dehiscent anthers form a cap over the stigma, starting pollen release (stage 12, Fig. 7b). Before anthesis, tubulin expression pattern differed between the two reproductive organs, with all genes similarly expressed in stamens, except for *LusTub7*, and a more variegated pattern in carpels. No significant change was observed in female organs before and after anthesis, while a sudden variation in steady state mRNA level took place in anthers containing mature pollen, with up-regulation of *LusTub 3 and* 4, paralleled by down-regulation of *LusTub2* and *5*, whose transcript become barely detectable (Fig. 7c). High standard deviation values among biological replicates likely reflect the consequence of an imperfect matching between asynchronous onset of anthesis, variable times of flower collection and the rapid changes in gene expression during transition from pA and A (24 h).

### Expression of duplicated β-tubulin genes

**Fig. 8:**
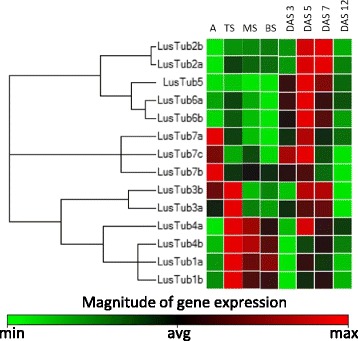
Clustergram of differential β-tubulin gene expression in stem sections and hypocotyls. Color ranges from green to red, through black, according to the manitude of relative gene expression, as shown in the scale bar. Targets are clustered according to their similarity in the expression pattern

It is generally accepted that duplicated genes in multicellular organisms are free to accumulate mutations resulting, over the time, in differential expression and eventually in subfunctionalisation, neofunctionalisation or nonfunctionalisation [51, 52]. Conversely, it has been most recently suggested that conservation of duplicated fundamental genes can be favoured by selection thanks to its buffering effect, in order to cope with accidental, non-functional mutations [53]. In flax we faced a very high level of retention of duplicated β-tubulin genes after the Whole Genome Duplication (WGD) event occurred during speciation, with just one probable gene loss, that of the putative *LusTub5* counterpart. As reported before, four out of six sets of the flax duplicated β-tubulin genes do not present any single aminoacid change. In accordance, when analysing ohnologs by pairwise alignment of their nucleotide sequence, we found very high nucleotide identity, 96.7 on average (between 91 and 98%), within coding sequences but a higher divergence, as expected, in transcribed noncoding regions (introns and 3’UTR), 81% on average, including gaps. However, there was a large variability in the identity degree among ohnolog pairs in these regions that may contribute to the regulation of gene expression, which ranged from 60 to 91%, as detailed in Table 2. We also compared portions of the genomic region upstream of the start codon, 0.5 and 1 kb long, respectively, which included the leader sequence and the putative promoter regions. Their degree of identity decreased with the distance from the start codon and was again largely variable among ohnolog pairs. While *LusTub2a* and *2b* retained striking nucleotide identity (85.9% over 1 kb), *LusTub4a-b* and *LusTub7a-b* diverged widely, showing only a 51–52% identity. Analysing our qPCR data by target, we observed that sibling genes have superimposable expression patterns both in the stem and hypocotyl, as shown by the clustergram in Fig. 8. The clustergram shows the data in a hierarchy, based on the degree of similarity of expression for different targets and samples. The most diverging pattern was that of *LusTub4a* and *4b*, possibly reflecting the greater divergence of their transcribed and non-transcribed regulatory sequences.

**Table 2:** Percent nucleotide identity between ohnologs

Gene	Transcribed regions						
	Coding		Non coding			Upstream regions	
	ORF	(AA)	intron1	intron2	3’UTR	0.5 kb UP	1 kb UP
1a/b	96.4	0	83.5	91.5	75.6	86.4	56.4
2a/b	97.7	0	90.8	98.4	83.3	86.8	85.9
3a/b	98.2	0	83.9	85.8	90.9	81.0	77.0
4a/b	96.3	0	78.9	60.0	73.5	54.1	51.5
6a/b	96.4	5	88.3	75.0	72.4	90.00	81.6
7a/b	95.3	3	87.1	85.0	57.3	57.6	52.5
7a/c	95.0	3	–	–	62.4	–	–
7b/c	91.0	2	–	–	84.1	–	–
Mean	96.72		85.42	82.62	75.50	75.98	67.48
SD	2.21		4.21	13.52	11.25	15.90	15.70

*ORF* Open reading frame, *AA* different aminoacids, *UTR* untranscribed region

## Discussion

The most evident feature of the flax β-tubulin gene family, based on cDNA sequence alignment, is the presence of five pairs of duplicated genes (*LusTub1*, *2*, *3*, *4* and *6*), plus one triplet, likely due to a further single-gene duplication (*LusTub7*), and one singleton (*LusTub5*). This configuration is in agreement with data from the flax genome assembly, inferring the occurrence of a WGD in the flax lineage, dated around 5–9 MYA [37]. This evidence is also supported by karyotype studies that assigned larger chromosome sets (2n = 30) to both *L. usitatissimum* and *L. bienne*, the likely wild ancestor of cultivated flax, with respect to other close relatives such as *L. grandiflorum* (2n = 16) [54]. This supports the occurrence of chromosome repatterning and diploydisation following tetraploydisation. The high level of retention of duplicated β-tubulin genes after the WGD event, with just one probable gene loss, the ohnolog of *LusTub5*, is in accordance with similar occurrence in other plants. Tubulin gene family expansion following genome duplication have been reported for the genome of cotton (*G. hirsutum*), a quite recent allotetraployd (1MYA) accounting for at least 19 β-tubulin genes [25], as well as for the *Populus* and *Salix* genera, both having 10 pairs of highly similar β-tubulin genes [23, 55], reminiscent of the ancient salicoid WGD event, estimated 60–65 MYA [56]. In the flax genome, gene expansion was reported for other gene families, such as β-galactosidase, cellulose synthase and chitinase-like [6, 8, 57], but duplicate gene retention was lower and characterized by selective gene losses and differential expansion of specific subfamilies. Our observation is supported by recent models of duplicated gene evolution suggesting that rates of duplicate gene retention vary among functional groups. Duplicate genes retained after WGD have the tendency to persist and proliferate following subsequent duplication events, while singleton showed an opposite tendency to be repeatedly restored to the original status [53]. The tendency to high retention rates was found to be more pronounced among genes encoding long complex proteins and proteins with high number of interactions. This is consistent with the gene balance hypothesis, which predicts that the fate of duplicate genes largely depends on maintaining a stoichiometric balance among members of macromolecular complexes [58, 59]. Such explanation is particularly suited for tubulins, whose unbalanced synthesis may induce lethal phenotype [60]. Duplicated genes are not only retained but were found to accumulate mutations at a slower rate than singleton genes in rice subspecies and *A. thaliana* ecotypes [53]. In the genome of *Populus*, relative rates of non-synonymous substitution were actually lower for genes with paralogs from the salicoid and eurosid whole-genome duplication events than for genes with no paralogs [56].

Tubulins in particular are very ancient, highly conserved proteins, since even single AA substitutions in a single isotype have high chance to confer lethal or dominant negative phenotypes which may severely alter growth morphology in plants [41] or lead to serious neurodevelopmental diseases, defined as tubulinopathies, in humans [61]. Therefore, strong purifying selection is likely to act toward the fixation of tubulin duplicates. Passive forces rather than adaptive mechanism are likely to act to keep tubulin gene family large. Nevertheless, tubulin genes can slowly evolve and acquire slightly different properties. In flax, aminoacid substitutions between tubulin ohnologs were minimal, up to 4–5, and clustered in the CTT. Although protruding from the microtubule core, this highly polymorphic domain can provide distinctive features to the proteins. It is in fact the binding site for a large set of microtubule associated proteins (MAPs; [62]) and the target for multiple post-translational modifications such as tyrosination, phosphorylation or glutamylation, as largely documented by immunological data (reviewed in [63]), although mass spectrometry-based analysis has recently questioned the abundance of such modifications in some plants or plant tissues [64, 65]. Although not affecting dimer formation, CTT is also a key determinant for microtubule assembly, influencing the kinetics of polymerization/depolymerisation [66]. Therefore, mutations in this domain can be well tolerated and may gradually accumulate providing slightly different properties to tubulin isotypes and to their corresponding microtubules.

Not only duplicated flax β-tubulin genes show minimal sequence divergence, but they also retain the same expression pattern, with some little bias toward one other or the other ohnolog observed in few cases, in the tissues we investigated. *LusTub4a* and *LusTub4b*, showing the higher, although modest in absolute values, difference at transcriptional level encode for identical proteins, but carry divergent regulatory regions. This suggests that differential expression may precede protein modification in tubulin gene evolution. In the aspen genome, in which the last whole genome duplication event dates back to 60–65 MYA, the ten pairs of duplicated β-tubulin genes still retain similar expression patterns, with few exceptions (i.e*PtrTub 9* and *PtrTub10*), despite a greater divergence of their aminoacid sequences [23]. A more detailed study of duplicated tubulin genes in different plants could reveal new interesting insights on the evolution mechanism of this fundamental gene family. The functional significance of the presence of numerous slightly different tubulin isoforms in plants is still poorly understood, despite the many attempts of assigning them functions.

Model systems, characterized by synchronously developing cells, may help to unravel this matter. Mesophyll cells in barley leaf sections have been used to study the differential and gradual modulation of steady state mRNA level of the five alpha tubulin genes that occur in parallel to the formation of differently oriented cortical microtubule arrays [67]. The cotton fibre, a trichome elongating from a single seed epidermal cell, is another useful model used for the identification of tubulin genes related to cell elongation and cellulose deposition [24–26]. One of these studies also led to the isolation of a specific cotton β-tubulin gene able to induce abnormal elongation in fission yeast [26].

Flax stems and hypocotyls have been successfully used as a model to identify transcriptional changes that might correlate to the different developing stage of xylem and phloem fibres [6, 9, 45]. Since secondary cell wall deposition in xylem fibres begins early in hypocotyl development and in the top section in the stem, it was expected that a substantial portion of the differentially transcribed genes detected would have mainly reflected phloem-specific developmental events. Microarray analysis showed that actin, tubulin and other cytoskeletal proteins are enhanced in developing fibres, but no information was reported about differential expression.

In this work, we observed that the relative level of β-tubulin transcripts was modulated during stem and hypocotyls elongation. Using the apex as a control, we observed a more or less pronounced up-regulation of most genes in the TS, which is characterised by an extensive coordinated growth of fibre cells with other cell types. This process is associated with a transverse orientation of cortical MT that assist the correct positioning of CESA complexes in the membrane. Some of the tubulin genes, *LusTub3*, *5* and *6*, were clearly down-regulated in the next stem sections, MS and BS, characterised by cortical MT re-orientation, concomitant with the arrest of elongation and gradual cell wall thickening in phloem fibres, while only cambium derivatives are still elongating. These two sections showed similar tubulin expression profiles, in accordance to a more general picture also envisaged by microarray analysis [6]. Conversely, up-regulation of *LusTub1*, *2* and *4* in TS was stably maintained across the stem. *LusTub7* paralogs showed an opposite behaviour, being mostly expressed in the apex and down-regulated in all the other sections. Interestingly, aspen *PtrTub1* and *PtrTub2*, the most expressed β-tubulin genes in the apex, belong to the same class II as *LusTub7*. Comparison of large expression datasets in different plants could provide hints for some functional difference among tubulin classes. In the hypocotyl, a generalized increase in tubulin gene expression was observed at DAS5 with respect to DAS3, which paralleled the gradual transition from cell fibre elongation to cell wall thickening, as documented by the *LuBGal1* expression. Similar to what observed in the stem, *LusTub3*, *5* and *6*up-regulation was transient and rapidly declined with fibre development, while the increase of *LusTub4* mRNA was more stable, and *LusTub1* and *2* transcript level dropped at DAS12. *LusTub7* paralogs were gradually downregulated with respect to early stages. Similarity in the relative expression pattern between stem and hypocotyl is in accordance with their correlation to fibre development. This could be further confirmed by expression studies on isolated fibres. How the different expression pattern observed influences microtubule array dynamics and cell function remains to be elucidated. Although post-transcriptional mechanisms are known to control tubulin levels, it is reasonable to infer that differential gene expression leads to the formation of different intracellular pools of tubulins, which may be further modified by post-translational modifications. It is then likely that microtubule arrays, randomly assembled from such different pools, have different kinetic properties and/or interact with different subsets of associated MAPs, making them better suited for specific functions (i.e, CESA positioning rather than vesicular transport for secondary cell wall deposition).

This can also apply to what observed in flax anthers. Complete pollen maturation at anthesis brings about changes in the steady-state transcript level of β-tubulin, with significant up-regulation of *LusTub 3* and *4*, paralleled by down-regulation of *LusTub2* and *5*, whose transcript become barely detectable. The newly synthesized tubulin pool might be more suited to build MTs more specialized for fine delivery of vesicles carrying proteins/lipids at the tip membrane to sustain polarized pollen tube growth.

## Conclusions

We first described the flax β-tubulin gene family as composed by fourteen expressed members, reminiscent of the last WGD event during flax speciation. ML evolutionary tree including the complete set of β-tubulin genes of eight angiosperm species suggests that the multiple gene family originated by subsequent duplications of four ancestral genes. Gene expression analysis in stem, hypocotyl and flower suggest that different tubulin pools may originate MT with possible specialized functions.

Functional redundancy of tubulins has since now made difficult to dissect possible functions by simple knockout or overexpression experiments. Complete characterization of the gene family and an overview of the expression pattern during flax fibre elongation are the pre-requisite for further studies aimed to elucidate functional differences among similar tubulins. Further confirmation of the observed expression pattern can be obtained in isolated fibres. Genome editing studies with CRISPR-Cas9, may reveal new insights on such tubulin specificities. A future goal could be the modulation of the expression of tubulin genes specifically involved in cell elongation, in order to increase fibre length in flax. Tubulin genes that are up- or down-regulated during fibre elongation, such as *LusTub3*, *5* and *6*, are good candidate for further studies.

## Additional files

Additional file 1: Table S1. PCR primers used for β-tubulin cloning. (DOCX 16 kb)

Additional file 2: List of the Genbank accession numbers of the aminoacid sequences used for the phylogenetic analysis of Fig. 2. (DOCX 18 kb)

Additional file 3: Table S2. qPCR primers. (DOCX 656 kb)
