# Supplementary material for: Evolutionary characterization and transcript profiling of β-tubulin genes in flax (Linum usitatissimum L.) during plant development
Source: BMC Plant Biol. 2017 Dec 8;17:237. doi: 10.1186/s12870-017-1186-0 (PMC5721616; doi:10.1186/s12870-017-1186-0)
Supplement: Supplementary file 1 — PCR primers used for β-tubulin cloning. (DOCX 16 kb) [file 12870_2017_1186_MOESM1_ESM.docx]

# Additional file 1

| Table S1 - PCR primers used for β-tubulin cloning | |  |
| --- | --- | --- |
| Primer Name | 5'-3' sequence | nt |
| LusTub1a_RSP | TAAACATAACATTGGGGTATTTCAG | 25 |
| LusTub1b_RSP | TTTGATACCCAGTTTCATTTCAGT | 24 |
| LusTub2a_RSP | CCGAAATAGGCAAAATAAATGAAAC | 25 |
| LusTub2b_RSP | ATGGCTAAAACTATATCACCGAGG | 24 |
| LusTub3a_RSP | AAGTAAAAGTAGGACACGATCCA | 23 |
| LusTub3b_RSP | AGCAAAGTACTAGGATATGATCCA | 24 |
| LusTub4a_RSP | CATGGATCACAGAAAATACATACC | 24 |
| LusTub4b_RSP | CAAGTACAAAGCCACAAATCAAC | 23 |
| LusTub5_RSP | GAATTTAATTAATCAAATCCCAAAAC | 26 |
| LusTub6a_RSP | GCAGCAACAACATAGCAGATATC | 23 |
| LusTub6b_RSP | TCAAACGCATTCATTACATAAGATTC | 26 |
| LusTub7a_RSP | TAGAACTTGTTCGCCCACAGCT | 22 |
| LusTub7b_RSP | GTTCGTCCACAGCTAAAGGCAG | 22 |
| LusTub7c_RSP | CAAGAGATTGACTGAACAAAGTAGAT | 26 |
| MREI | ATGMGWGARATYCTYCAYRTYCA | 23 |
| Fex1 | AACTGGGCBAARGGNCAYTAYAC | 23 |
| Rex1 | ACCATRCAYTCRTCDGCRTTYTC | 23 |
| Fin2 | GARAAYGCHGAYGARTGYATG | 21 |
| Rin2 | CRAAVCCBACCATGAARAARTG | 22 |
| T7 (dT)_25_ | (dT)_25_ TAATACGACTCACTATAGGG | 27 |
