# Supplementary material for: Evolutionary characterization and transcript profiling of β-tubulin genes in flax (Linum usitatissimum L.) during plant development
Source: BMC Plant Biol. 2017 Dec 8;17:237. doi: 10.1186/s12870-017-1186-0 (PMC5721616; doi:10.1186/s12870-017-1186-0)
Supplement: Supplementary file 2 — List of the Genbank accession numbers of the aminoacid sequences used for the phylogenetic analysis of Fig. 2. (DOCX 18 kb) [file 12870_2017_1186_MOESM2_ESM.docx]

# Additional file 2

| Table S2 qPCR primers | |  |  |
| --- | --- | --- | --- |
| **Primer Name** | **5'-3' sequence** | **nt** | **Amplicon (bp)** |
| LusTub1a_Fw | CTGAATGGCTTCTTTGGGTCTACGTC | 26 | 151 |
| LusTub1a_Rv | AAATAGGAAGGAAATAAACATAACATTGGGG | 31 |  |
| LusTub1b_Fw | GTGTTTCTGTTGTGGTTTGCTTGGC | 25 | 95 |
| LusTub1b_Rv | AAGAACCCCCCACATTACATGTTTCC | 26 |  |
| LusTub2a_Fw | GAGCTTAGTGCTCCGGTCGACG | 22 | 188 |
| LusTub2a_Rv | CACCGAAATAGGCAAAATAAATGAAACAC | 29 |  |
| LusTub2b_Fw | TTATTGAGCTTAGTGTCCGGTCGACAC | 27 | 181 |
| LusTub2b_Rv | ATGGCTAAAACTATATCACCGAGGCAAA | 28 |  |
| LusTub3a_Fw | TTTCTGGGTCGAGAGTGGTGCC | 22 | 122 |
| LusTub3a_Rv | TCAGGCATTTAACAAAAGTAAAAGTAGGACAC | 32 |  |
| LusTub3b_Fw | GCTGCTGTTGTTTTTTTCATGTTCTCAG | 28 | 139 |
| LusTub3b_Rv | AGGCATTTAACAAAAGCAAAGTACTAGGATATG | 33 |  |
| LusTub4a_Fw | GAAGCTTTCTCTGGTTTGTGTTGGG | 25 | 105 |
| LusTub4a_Rv | GGATCACAGAAAATACATACCAAGCCAC | 28 |  |
| LusTub4b_Fw | GGGAAGGTATGGACGAGATGGAGTTC | 26 | 215 |
| LusTub4b_Rv | AAAGGGGGGAAAACAGACCATGC | 23 |  |
| LusTub5_Fw | GGACGACGATGAGGAGGAGATGG | 23 | 184 |
| LusTub5_Rv | CAATAATTCACGAACAAATTTCACCGG | 27 |  |
| LusTub7a_Fw | AGAGTATCAGTAGTCGTGCTTCGTTTCAAC | 30 | 164 |
| LusTub7a_Rv | ACTTGAAAATTACAGCACCATTGTTCTCC | 29 |  |
| LusTub7b_Fw | GTGGTGGTTTCCTTGTTATCTTTCTGTTG | 29 | 124 |
| LusTub7b_Rv | GTTCGTCCACAGCTAAAGGCAGTTG | 25 |  |
| LusTub7c_Fw | CAGGGAGAAGAAGAGTACGAGTAGTAGTTTTTG | 33 | 209 |
| LusTub7c_Rv | GCAAGAGAATCCAGTACAATTTAGACAGAAATTAG | 35 |  |
| LusTub6a_Fw | GGATTCGGCAAAGAAATGAATGAATG | 26 | 130 |
| LusTub6a_Rv | GCAACAACATAGCAGATATCAAAAGCATTC | 30 |  |
| LusTub6b_Fw | TAGTATTTGCCATTTGGGGATTGATTATATTC | 32 | 98 |
| LusTub6b_Rv | CGCATTCATTACATAAGATTCAATAGATCACAAC | 34 |  |
| GAPDH_Fw | AGGTTCTTCCCGCTCTCAAT | 20 | 138 |
| GAPDH_Rv | CCTCCTTGATAGCAGCCTTG | 20 |  |
| EF1A_Fw | GCTGCCAACTTCACATCTCA | 20 | 140 |
| EF1A_Rv | GATCGCCTGTCAATCTTGGT | 20 |  |
| LuBGAL1_Fw | GTTTCTGCTGATGCCACAGTAACA | 24 | 92 |
| LuBGAL1_Rv | TTTGCTAGGAACGCTGCACAC | 21 |  |
