# Supplementary material for: Evolutionary characterization and transcript profiling of β-tubulin genes in flax (Linum usitatissimum L.) during plant development
Source: BMC Plant Biol. 2017 Dec 8;17:237. doi: 10.1186/s12870-017-1186-0 (PMC5721616; doi:10.1186/s12870-017-1186-0)
Supplement: Supplementary file 3 — qPCR primers. (DOCX 656 kb) [file 12870_2017_1186_MOESM3_ESM.docx]

# Additional file 3

# List of the Genbank accession numbers of the aminoacid sequences used for the phylogenetic analysis of Figure 2

***Arabidopsis thaliana***

AtTub1: NP_177706

AtTub2/3: NP_568959

AtTub4:NP:_199247

AtTub5:NP:_564101

AtTub6: NP_196786

AtTub7:NP_180515

AtTub8: NP_568437

AtTub9: NP_193821

**Maize (*Zea mays*)**

ZmTUB1: P18025

ZmTUB2: P18026

ZmTUB3: Q43695

ZmTUB4: Q41782

ZmTUB5: Q43697

ZmTUB6: Q41783

ZmTUB7: Q4178

ZmTUB8: Q4178

**Rice (*Oryza sativa*)**

OsTUB1: Q43594

OsTUB2: Q8H7U1

OsTUB3: Q40665

OsTUB4: BAB39951

OsTUB5: P46265

OsTUB6: Q76FS3

OsTUB7: P37832

OsTUB8: Q76FS2

**Barley (*Hordeum vulgare*)**

Hvu Tub1: CAM58979

Hvu Tub2: CAM58980

Hvu Tub3: CAM58981

Hvu Tub4: CAM58982

Hvu Tub5: CAM58983

Hvu Tub6: CAM58984

Hvu Tub7: CAM58985

Hvu Tub8: CAM58986

**Cotton (*Gossypium hirsutum*)**

GhTub2, 4,8,10,11,13-19: EU375992–EU376003

GhTub1: AY345610

GhTUB3: AF521240

GhTUB5: AY345607

GhTUB6: AY345608

GhTUB7:AY345609

GhTUB9: AY345606

GhTUB12: DQ023526

**Black cottonwood (*Populus trichocarpa*)**

PtrTub 1: XM_002298294

PtrTub 2: XM_002313368

PtrTub 3: XM_002299505

PtrTub 4: XM_002304452

PtrTub 5: XM_002309073

PtrTub 6: XM_002323509

PtrTub 7: XM_002299006

PtrTub 8: XM_002330576

PtrTub 9: XM_002298000

PtrTub 10: XM_002304456

PtrTub 11: XM_002318483

PtrTub 12: XM_002321476

PtrTub 13: XM_002298418

PtrTub 14: XM_002313978

PtrTub 15: XM_002298419

PtrTub 16: XM_002313977

PtrTub 17: XM_002307924

PtrTub 18: XM_002322573

PtrTub 19: XM_002300667

PtrTub 20: XM_002307691

**Barrel clover (*Medicago truncatula*)**

XP_013457308.1

XP_003631111.1

XP_003630513.1

XP_003604818.1

XP_003604816.1

XP_003624926.1

XP_003603765.1

XP_003594596.1

XP_003592284.1

***Chlamydomonas reinhardtii***

P04690.1
